# Supplementary material for: From 2D to 4D: a Containerized Workflow and Browser to Explore Dynamic Chromatin Architecture
Source: bioRxiv. 2025 Jul 18:2025.07.13.664622. Preprint. [Version 1] doi: 10.1101/2025.07.13.664622 (PMC12338655; doi:10.1101/2025.07.13.664622)
Supplement: Supplement 2 — Figure S2: 4DGB on EDGE Screen capture of the 4DGB EDGE implementation, showing the list of existing jobs, which the user can navigate to view the final data products. [file media-2.pdf]

EDGE Bioinformatics

Not Secure bio-4dgb.lanl.gov/user/projectlist

New Chrome available

All Bookmarks

My Projects My uploads Job Queue

DR

- Home
- Public Projects
- Upload Files

WORKFLOWS

- 4DGB Workflow

Show all projects available to me

My Projects

Search

Drag headers here to group by

Project	Type	Status	Shared	Public	Created	Updated	Actions
monkey-01_with-tracks	4DGB Workflow	Complete	Yes	No	12/16/2023, 9:46:00 AM	12/16/2023, 12:22:59 PM	
monkey-01_no-tracks	4DGB Workflow	Complete	No	No	12/14/2023, 1:35:26 PM	12/14/2023, 1:51:17 PM	
A549_chrX_run-02_with_tracks	4DGB Workflow	Complete	Yes	No	12/13/2023, 1:38:14 PM	12/13/2023, 4:13:48 PM	
A549	4DGB Workflow	Complete	Yes	No	8/28/2023, 12:58:39 PM	12/13/2023, 2:04:25 PM	
A549_chr7	4DGB Workflow	Failed	No	No	8/30/2023, 3:34:54 PM	8/30/2023, 6:07:00 PM	
A549_chr7_with-tracks	4DGB Workflow	Failed	No	No	8/30/2023, 3:41:03 PM	8/30/2023, 5:31:00 PM	
A549_with_tracks	4DGB Workflow	Failed	No	No	8/29/2023, 4:26:44 PM	8/29/2023, 5:13:00 PM	
basic_test_with_tracks	4DGB Workflow	Complete	No	No	8/28/2023, 4:11:46 PM	8/28/2023, 4:26:00 PM	
basic_test	4DGB Workflow	Complete	No	No	8/28/2023, 4:08:41 PM	8/28/2023, 4:23:00 PM	
MRC5_03	4DGB Workflow	Complete	No	No	6/6/2023, 1:17:22 PM	6/6/2023, 1:29:00 PM	

10 rows 1-10 of 10

bio-4dgb.lanl.gov/user/projectlist
