# Supplementary material for: From 2D to 4D: a Containerized Workflow and Browser to Explore Dynamic Chromatin Architecture
Source: bioRxiv. 2025 Jul 18:2025.07.13.664622. Preprint. [Version 1] doi: 10.1101/2025.07.13.664622 (PMC12338655; doi:10.1101/2025.07.13.664622)
Supplement: Supplement 1 — Figure S1: 4DGB on EDGE Screen capture of the 4DGB Edge home screen, showing a capture of the tool instructions and both side and top menus for access to web-based capabilities. [file media-1.pdf]

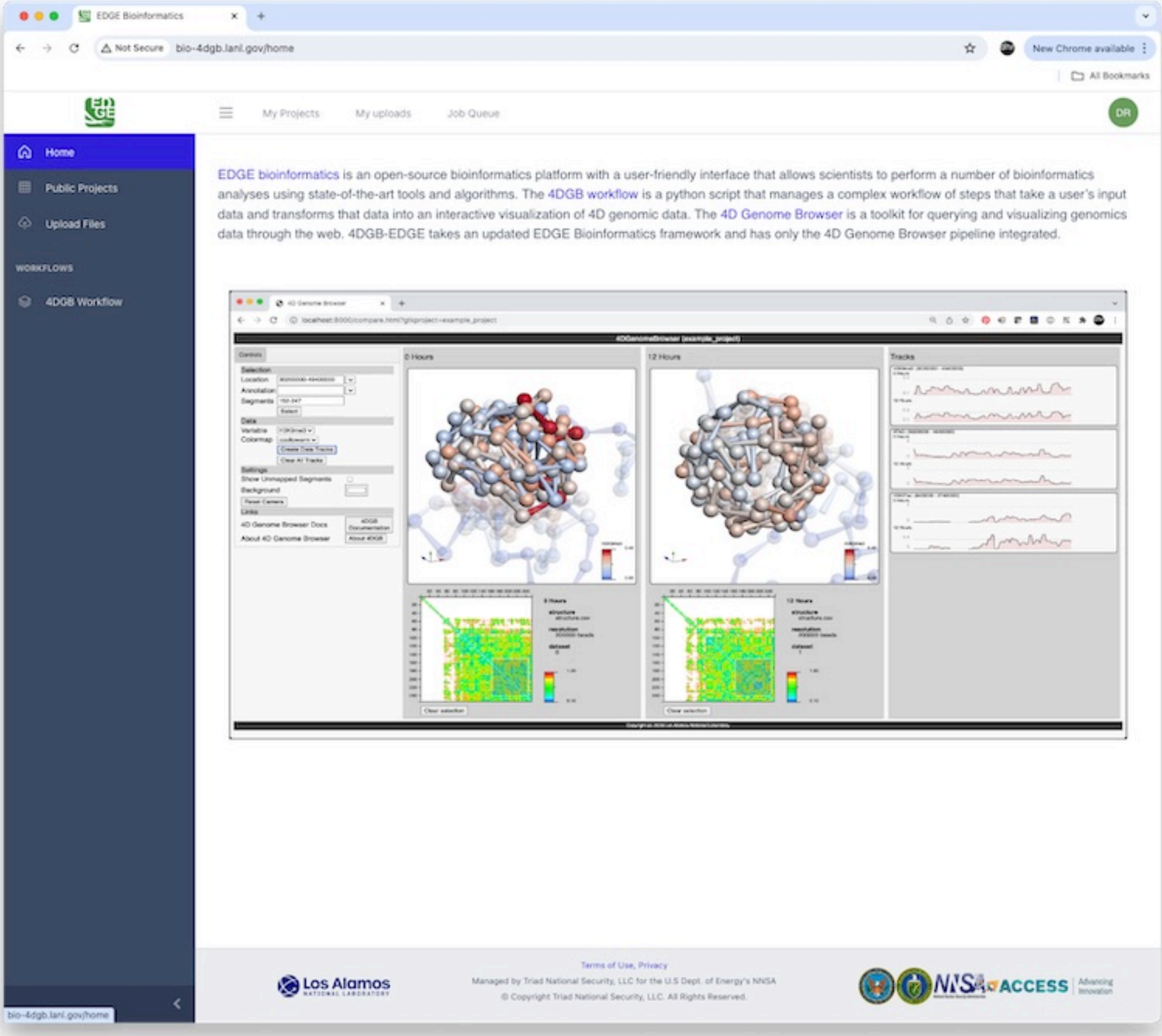
EDGE Bioinformatics
Not Secure
bio-4dgb.lanl.gov/home
New Chrome available
All Bookmarks
My Projects
My uploads
Job Queue
DR
Home
Public Projects
Upload Files
WORKFLOWS
4DGB Workflow
EDGE bioinformatics is an open-source bioinformatics platform with a user-friendly interface that allows scientists to perform a number of bioinformatics analyses using state-of-the-art tools and algorithms. The 4DGB workflow is a python script that manages a complex workflow of steps that take a user's input data and transforms that data into an interactive visualization of 4D genomic data. The 4D Genome Browser is a toolkit for querying and visualizing genomics data through the web. 4DGB-EDGE takes an updated EDGE Bioinformatics framework and has only the 4D Genome Browser pipeline integrated.
0 Hours
12 Hours
Tracks
Terms of Use, Privacy
Managed by Triad National Security, LLC for the U.S Dept. of Energy's NNSA
© Copyright Triad National Security, LLC. All Rights Reserved.
Los Alamos
ACCESS
bio-4dgb.lanl.gov/home
